# Marine ship instance segmentation by deep neural networks using a global and local attention (GALA) mechanism

**DOI:** 10.1371/journal.pone.0279248

**Published:** 2023-02-24

**Authors:** Zequn Sun, Chunning Meng, Tao Huang, Zhiqing Zhang, Shengjiang Chang

**Affiliations:** 1 Institute of Modern Optics, Nankai University, Tianjin City, China; 2 China Coast Guard Academy, Ningbo City, China; 3 717 Research Institute of China Shipbuilding Industry Corporation, Wuhuan City, China; 4 State Key Laboratory of Applied Optics, Changchun Institute of Optics, Fine Mechanics and Physics, Chinese Academy of Sciences, Changchun City, China; Kingston University, UNITED KINGDOM

## Abstract

Marine ships are the transport vehicle in the ocean and instance segmentation of marine ships is an accurate and efficient analysis approach to achieve a quantitative understanding of marine ships, for example, their relative locations to other ships or obstacles. This relative spatial information is crucial for developing unmanned ships to avoid crashing. Visible light imaging, e.g. using our smartphones, is an efficient way to obtain images of marine ships, however, so far there is a lack of suitable open-source visible light datasets of marine ships, which could potentially slow down the development of unmanned ships. To address the problem of insufficient datasets, here we built two instance segmentation visible light datasets of marine ships, MariBoats and MariBoatsSubclass, which could facilitate the current research on instance segmentation of marine ships. Moreover, we applied several existing instance segmentation algorithms based on neural networks to analyze our datasets, but their performances were not satisfactory. To improve the segmentation performance of the existing models on our datasets, we proposed a global and local attention mechanism for neural network models to retain both the global location and semantic information of marine ships, resulting in an average segmentation improvement by 4.3% in terms of mean average precision. Therefore, the presented new datasets and the new attention mechanism will greatly advance the marine ship relevant research and applications.

## 1 Introduction

Image segmentation plays an essential role in many visual understanding and object detection systems [1–3]. It involves a process that employs the intensity (brightness) or other information (e.g., edge) of an image to divide the image into independently connected regions. Image segmentation algorithms can be classified into at least two categories, i.e., semantic segmentation and instance segmentation. Semantic segmentation performs pixel-level labelling using a set of colors (object categories), while instance segmentation extends semantic segmentation by simultaneously detecting and delineating each object of interest in an image [3, 4]. Compared to object detection which merely detects the location of an object and places a window over it, instance segmentation performs more like a combination of object detection and semantic segmentation that not only detects the locations of all specific objects but also outlines and classifies individual detected objects [5]. As to marine ship segmentation, semantic segmentation classifies all ships in an image into one category, by labelling all ships with one color, while instance segmentation detects individual ships and classifies them into different categories. The applications of instance segmentation have been launched successfully in scenarios such as unmanned vehicle development [6, 7], human-computer interaction [8, 9], bio-medicine development [10–12], video surveillance [13–15], and marine ship monitoring [16–18].

Since marine ships are the vehicle of ocean-related activities such as marine scientific research and education, transoceanic transport and marine fishing industry, image and video analysis of marine ships including instance segmentation of marine ships has received an increasing attention in the past years [18–20]. Instance segmentation of marine ships is capable of providing important information such as the relative location of a ship with respect to other ships or surrounding obstacles, which is crucial for ship travel safety. Nowadays, various types of imaging techniques such as radar and infrared camera, have been equipped on a modern marine ship or an intelligent unmanned ship [16]. The spatial analysis of the ship imaging data can provide accurate environmental information to assist the ships to autonomously avoid crashing with other ships and natural obstacles in the ocean. Moreover, by segmenting a ship with respect to the background that may contain spatial information (for example, a known island or city), we can obtain the identity of the ship and the absolute location of the ship on earth. Therefore, instance segmentation of marine ships from a complex maritime background is essential for many ocean-related activities.

At present, satellite, synthetic aperture radar (SAR), infrared imaging (IR) and visible light (VL) imaging, are the main imaging tools to record marine ships, resulting in several different types of open-source databases for marine ship segmentation. These databases include satellite remote sensing images [21–23], SAR images [24, 54], IR images [25–27], and VL images [28]. The satellite images usually have a very large field of view, covering a wide space, but their image resolution is low, providing no accurate information (e.g. the shape and type) of a ship. SAR imaging can perform regardless of weather conditions, but SAR images usually contain a large amount of scattering noise and do not have rich spectral information, which is not convenient for subsequent ship segmentation purpose.IR imaging has strong penetration capability and is not easily affected by environmental conditions, but the contrast and signal-to-noise ratio of the obtained IR images are usually not high enough, resulting in a lack of color and texture information of ships. In contrast, VL images have many unique advantages over other types of images such as high resolution, the inclusion of color and texture information, high signal-to-noise ratio, high contrast and of rich details, and thus VL images can be a strong complementary portion to satellite, SAR and IR images [16]. With these merits, VL images can provide clear information of ship features (e.g., shape) that are crucial for subsequent ship detection, segmentation and classification. In addition, VL images can be easily obtained in a very low-cost way by using our routine cell phones and cameras, and therefore, they are suitable to build applications that need large scale data. However, so far, there are only a limited number of open-source databases for VL marine ship images. Although two VL image datasets of marine ships were presented by Zhang et al. and Sun et al. respectively [16, 18], these datasets have not been made publicly available, the scale of these datasets is relatively small and the labelling of the datasets in terms of ship categories is not fine enough.

The instance segmentation of marine ships in VL images is a challenging image processing task. In general, the existing segmentation methods can be mainly classified into thresholding [29], segmentation based on edge [30], region [31], super-pixel [32, 33], correlation theory [34, 35] and deep learning [36, 37]. The deep learning approach has gained increasing attention recently, and a challenge of instance segmentation based on deep learning is the acquisition of the location and semantic mask of each instance. Mask R-CNN implemented a general framework that can efficiently detect objects in an image while simultaneously generating a high-quality segmentation mask for each instance, which extends the Faster R-CNN model by adding a branch to predict an object mask in parallel with the existing branch for bounding box recognition [5]. Since the two-stage instance segmentation approach has high accuracy but suffers from low speed, the single-stage instance segmentation approach has been proposed to improve the segmentation efficiency. BlendMask was proposed to achieve improved mask prediction by using an effective combination of instance-level and semantic information with lower-level fine-granularity [36]. PolarMask was proposed to formulate an instance segmentation problem as the prediction of the instance contours through instance center classification and dense distance regression in a polar coordinate system, providing a new way of designing mask contours [38]. CenterMask is a single-stage anchor-free instance segmentation method that designed a new spatial attention-guided mask branching [37]. Different from the abovementioned approaches that rely on accurate edge detection, models such as SOLO and SOLOv2 can directly segment instance masks and learn instance mask labels, enabling end-to-end optimization [39, 40]. The SOLO algorithms were demonstrated that they could outperform both the two-stage and one-stage algorithms. Instance segmentation of marine ships in VL images has also attracted increasing attention in the field. Zhang et al proposed an integrated ship segmentation method based on discriminators and extractors to reduce the interference factors of complex ocean backgrounds [18]. To preserve the global information of a ship, Sun et al proposed a method using precise RoI pooling and global mask head that could improve the performance of ship instance segmentation [16]. showing that the use of the global and complete appearance information of a ship can increase the performance of instance segmentation. However, this method is too complex due to the extensive modification in the network architecture, and there is still some room for further improvement by the preservation of both the global and local ship information, while it is not trivial for the existing deep learning models to effectively retain both the global and local information.

The human attention mechanism can be a potential solution to retain both global and local information. In a complex scene, human attention can be attracted easily by salient features and regions. Inspired by this observation, the attention mechanism was introduced into computer vision. The use of an attention mechanism in instance segmentation can guide the segmentation to the most important regions of an image and ignore irrelevant parts [41]. The attention mechanism amplifies the role of key feature maps by assigning them greater weights. It is also important to note that the attention mechanism is a plug-and-play module that can be efficiently plugged into many deep learning models, which leads to a great success in the fields such as image classification, object detection, semantic segmentation, instance segmentation and 3D vision [42–51]. In the existing deep neural networks, e.g. SENet and CBAM [42, 43], the attention mechanism was mainly used to convert the 2D feature maps into pixel feature maps by dimensionality reduction via 2D global pooling for feature map weight recalibration. These models using 2D global pooling mainly emphasized on the global information while ignored the local information. In contrast, the one-dimensional strip pooling is able to retain merely the local information along the spatial direction accurately [52]. Therefore, the combination of 2D global pooling and 1D strip pooling is a promising approach to preserve both the global and local information of an image.

In this work, in order to meet the urgent demand for open-source VL databases of marine ships, we collect and label two VL marine ship datasets, and test the segmentation performance of several existing deep learning models on our VL datasets. Moreover, we further propose a global and local attention (GALA) mechanism to improve the performance of the existing instance segmentation models, by combining 2D global pooling and 1D strip pooling to retrain both the global and local feature information. Both the datasets and the proposed approach are open-source available together with this study.

## 2 Methods

### 2.1 Analysis of the existing ship-related databases

**Fig 1:**
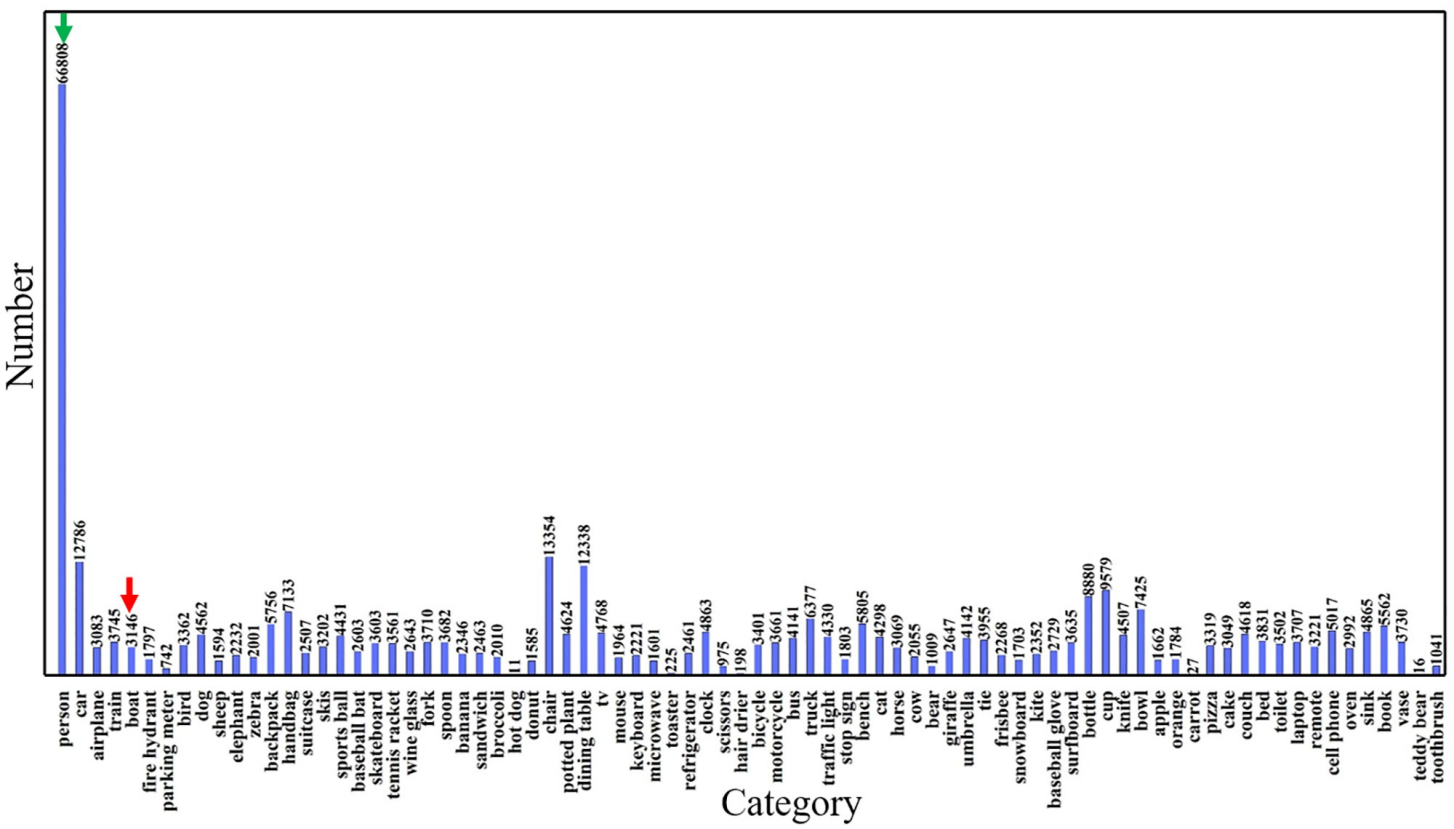
Distribution of the numbers of individual categories in the MS COCO dataset. The dataset has a total number of 80 categories, where the green and red arrows represent the number of images in the ‘person’ and ship category respectively.

To investigate whether the existing databases of marine ships contain sufficient VL images that can be used for marine ship instance segmentation, we first explored the open-source datasets, covering several image types, reported in previous studies (as shown in Table 1). These datasets included the VL dataset Sea Ships [53], the IR dataset Distant sea ships [54], the SAR dataset SAR-Ship-Dataset and SSDD [55, 56], the SAR dataset HRSID [57], and the MS COCO dataset that contains VL images of marine ships [58]. Sea Ships, which contains 31455 VL images and covers six commonly seen ship types, is mostly used for the object detection task. The Distant sea ships dataset consists of merely 3132 images of the long-wavelength IR type. The SAR-Ship-Dataset and SSDD datasets are only composed of high-resolution SAR images. In addition, these datasets are mainly designed for object detection purpose, by placing a window on a ship detected, and they do not have annotations required for instance segmentation purpose which needs to label the location, shape, and category of individual ships. HRSID can be used for ship instance segmentation because it contains the annotations required for instance segmentation, but it is of SAR type. The MS COCO dataset is a large open-source dataset commonly used for instance segmentation. To investigate whether this dataset contains specific VL images of marine ships that can be used for instance segmentation, we conducted a detailed analysis of the names of all images contained in the MS COCO dataset, which could indicate the contents or categories (person, car, boat, and so on) presented in the images. Fig 1 shows the number of individual categories in the MS COCO dataset, indicating that the distribution of the quantities of individual categories is uneven. The ‘person’ category takes up to 54% of the total number of images, while only about 2% of the images belongs to the ‘ship’ category.

**Table 1:** Overview of several existing open-source datasets of marine ships.

Dataset	Type	Task	Images	Size of images
Sea Ships [53]	VL	Object detection	31455	1920×1080
Distant sea ships [54]	IR	Object detection	3132	640×512;320×256
SAR-Ship-Dataset [55]	SAR	Object detection	43819	256×256
SSDD [56]	SAR	Object detection	1160	190-526×214-668
HRSID [57]	SAR	Object detection and segmentation	5604	800×800
MS COCO [58]	VL	Object segmentation	3146	–

**Fig 2:**
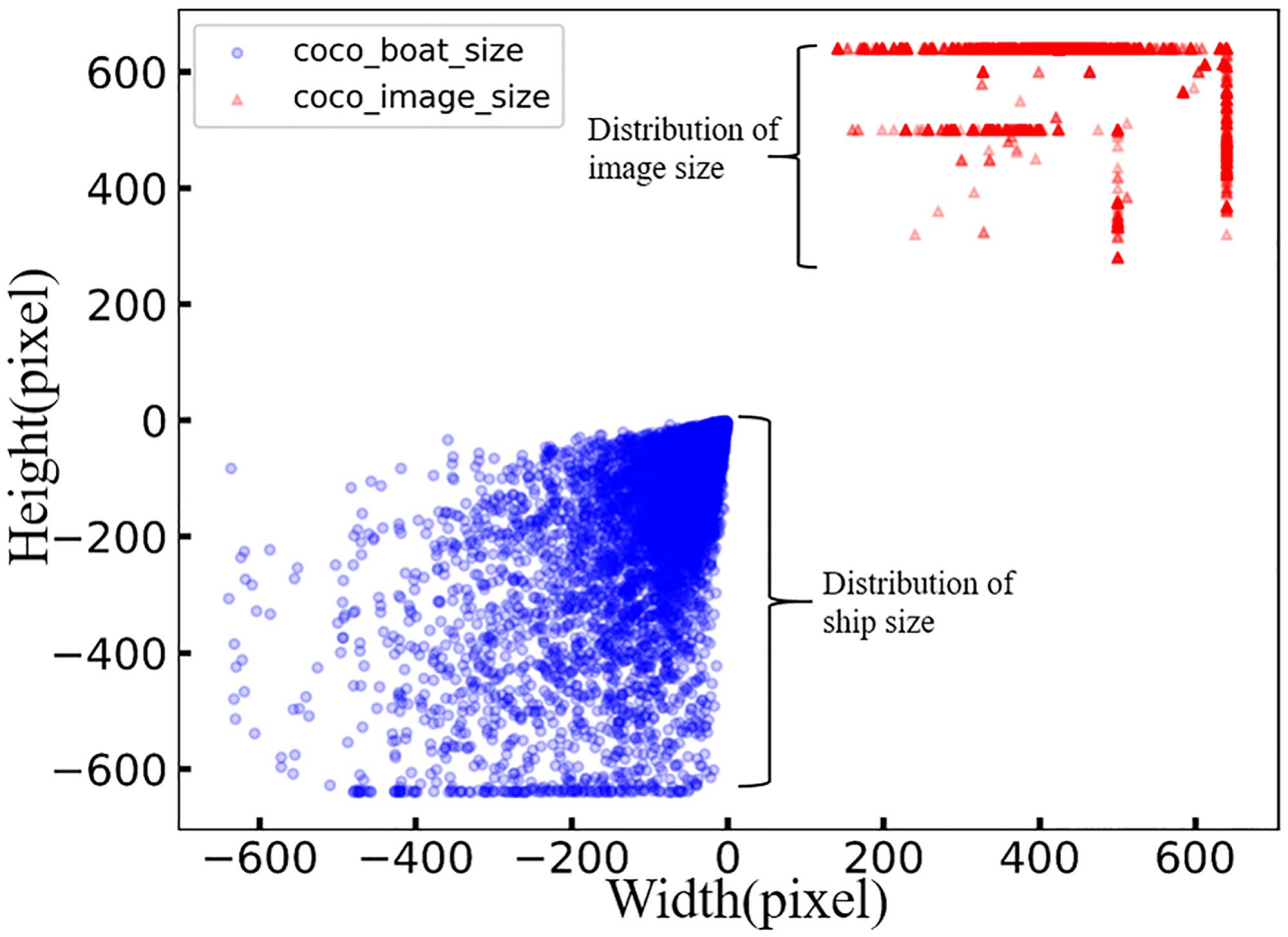
The distribution of image and object sizes for the MS COCO ‘ship’ category. A red triangle in the plot indicates the size of an image. A blue circle indicates the size of a ship.

We then developed an image extraction script to extract the VL images of marine ships from the MS COCO dataset, and we named this subset as coco_boats. Since the image size and object scale in an image are important factors that can affect the performance of an algorithm, we also analyzed these two factors of images in coco_boats (Fig 2). We found that the length and width of the images are clustered at 500 and 650 pixels (red, Fig 2), which are evenly distributed in a line, while the length and width of the ships are located within [50, 650] and [50, 450] respectively (blue, Fig 2). This analysis shows that the image size and ship scale are not diverse enough. Taken together, IR images and SAR images took up most of the open-source marine ship datasets, while currently there is a lack of open-source VL datasets of marine ships that can be used for instance segmentation, and we extracted the coco_boats dataset from MS COCO to facilitate the research of marine ship instance segmentation.

### 2.2 Two new datasets of VL images for marine ship instance segmentation

A reason of insufficient open-source VL datasets for marine ship instance segmentation may be the difficulty in data collection and the relatively time-consuming and laborious nature of labelling segmentation data. To overcome the problem of insufficient datasets, we developed a script to collect images from the ‘Google Image’ platform, using ‘ship’ as the searching keyword, after which we manually selected the true marine ship images and labelled the segmentations of ships using the LabelMe software [59]. Different from placing a window over a ship used for object detection purpose, which is commonly used in the object detection field, labelling ships for instance segmentation purpose is a very time-consuming task. In this labelling procedure, we first drew a polygon mask of a ship by following and marking the shape of the ship in an image without interruption, and then we named and classified the labelled ship. After delineating all the ships in an image with polygonal annotations, we generated an image annotation file using the json format, which will be later fed into a neural network. In total, we labelled a number of 6.2k images, which took about 400 hours. Note that the naming and annotation methods of the new datasets are in consistent with those of MS COCO.

From the Google Image platform, we generated two types of marine ship instance segmentation datasets, named as MariBoats and MariBoatsSubclass respectively, which we hoped can be used for different research purpose. The MariBoats dataset used all the 6.2k images and all the labelled ships were assigned to only one category, namely ‘ship’, resulting in 15.7k ship segmentation annotations. Compared with MariShipInsSeg [16], our dataset has a higher number of images and is open-source. This dataset with one category can satisfy the basic instance segmentation requirements (For example, avoiding obstacles (ships) during unmanned driving in the complex sea scene). To obtain a finer distinction of the labelled ships, we built MariBoatsSubclass, containing 3.1k images and 4.5k ship annotations. This dataset has six categories of marine ships: Engineering Ship (Eng.), Cargo Ship (Carg.), Speedboat (Sp.), Passenger Ship (Pass.), Official Ship (Off.), and Unknown Ship (Unk.). This dataset can be used for both the ship instance segmentation and the precise identification of marine ship categories in marine scenes.

#### 2.2.1 MariBoats

**Fig 3:**
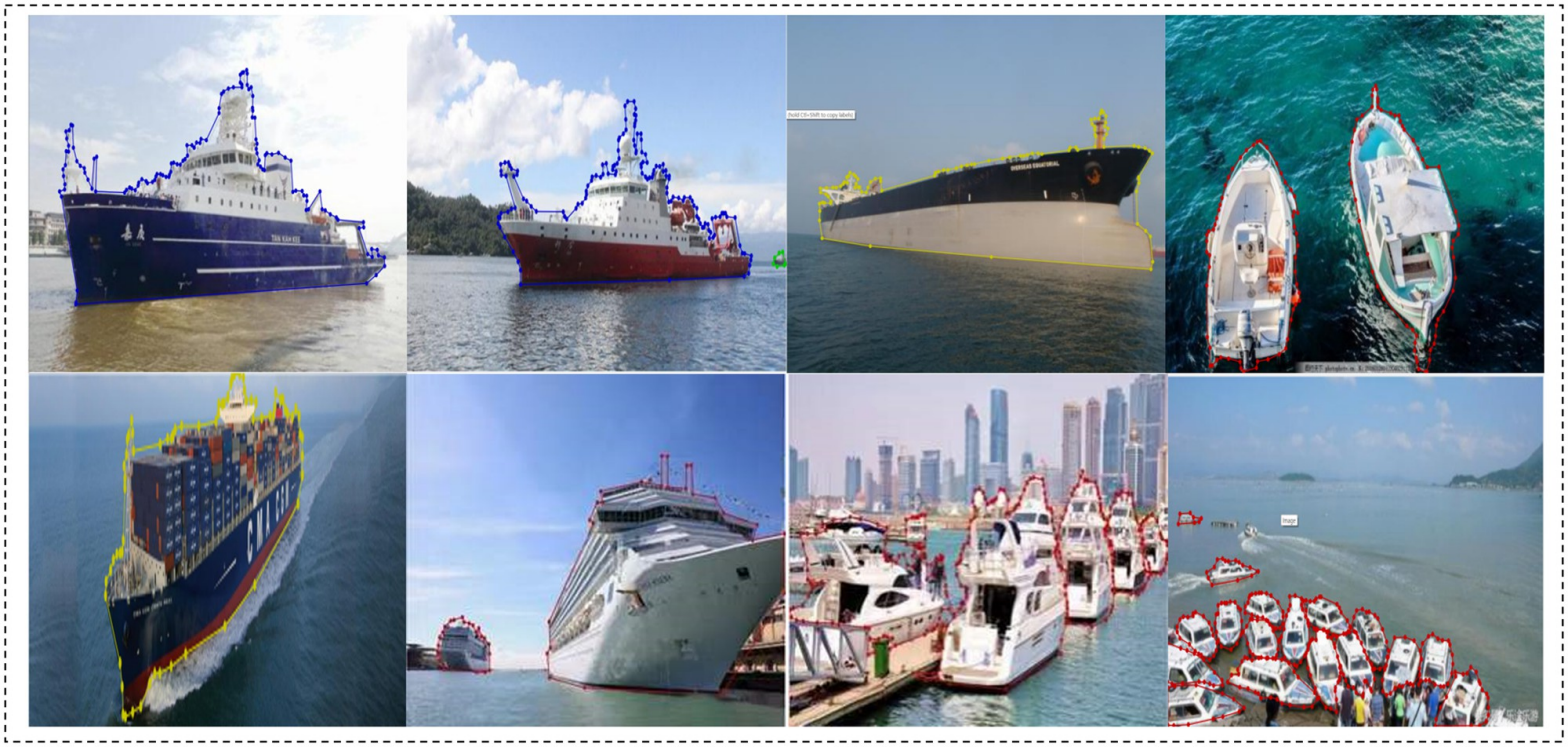
Representative images from MariBoats with manual annotations. The manual delineations are indicated in blue, yellow or red.

The MariBoats dataset is comprised of 6,271 ship images and 15,777 ship segmentation annotations, having only one category. The images in this dataset were partially extracted from 13717 ship images searched on ‘Google Image’ using keywords such as cargo ships, fishing boats, etc. We excluded those images of low quality, blurred, misrelated to ships, and the ones with duplicate content. We also included the coco_boats dataset (the subset of the MS COCO dataset containing VL images of ships) into MariBoats. Fig 3 shows our delineations of individual ships in representative images of MariBoats, illustrating the laborious nature of the delineation work. Avoiding the work of repeating such delineations is the motivation to make our datasets publicly available.

**Fig 4:**
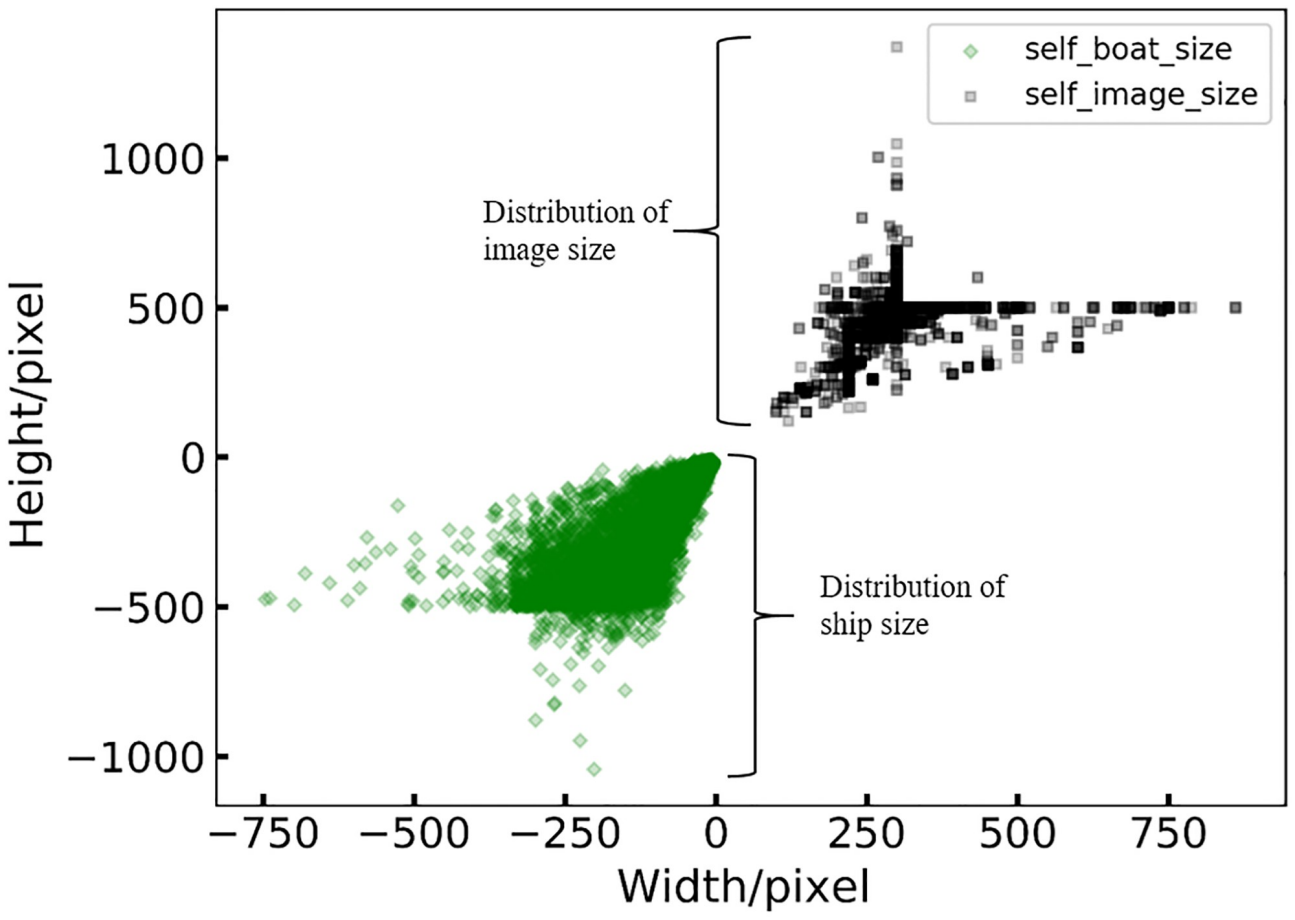
The distribution of image and ship sizes in the self_boats dataset.

**Fig 5:**
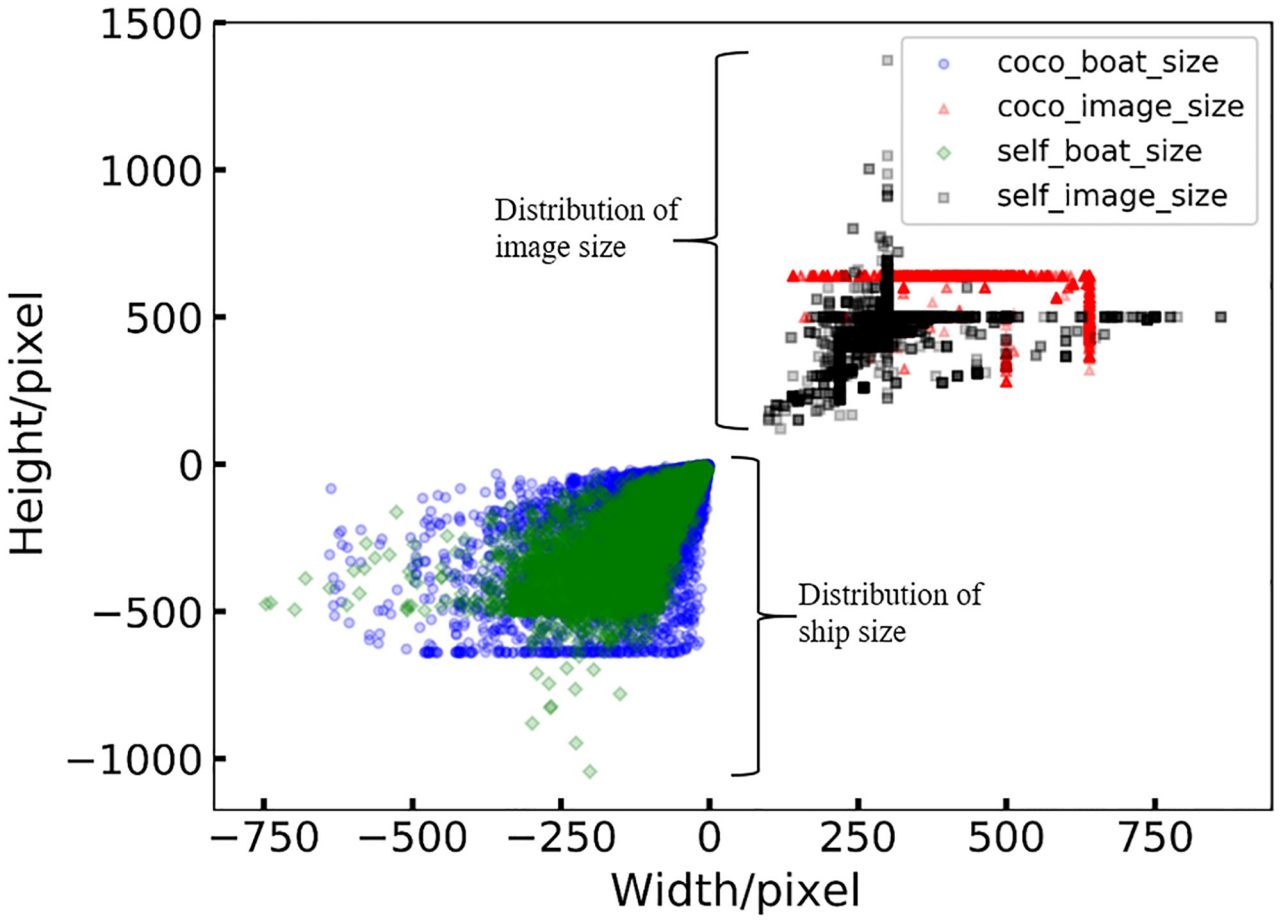
The distribution of image and ship sizes in the MariBoats dataset. It is a combination of Figs 2 and 4.

To distinguish the images collected from Google Image with coco_boats, we named the former as the ‘self_boats’ dataset. The distribution of the image size and ship scale of self_boats is shown in Fig 4. Compared with coco_boats (Fig 4), the image size distribution (black, Fig 2) of the self_boats dataset is located within [50, 800] in length and [50, 750] in width, and the size of ships (green, Fig 4) is distributed within [50, 550] in length and [50, 500] in width. From the scatter plots of image size and ship scale distribution of MariBoats, which is a combination of self_boats and coco_boats (Fig 5), we see that the image size and ship scale of the MariBoats dataset are more diverse than coco_boats.

#### 2.2.2 MariBoatsSubclass

**Fig 6:**
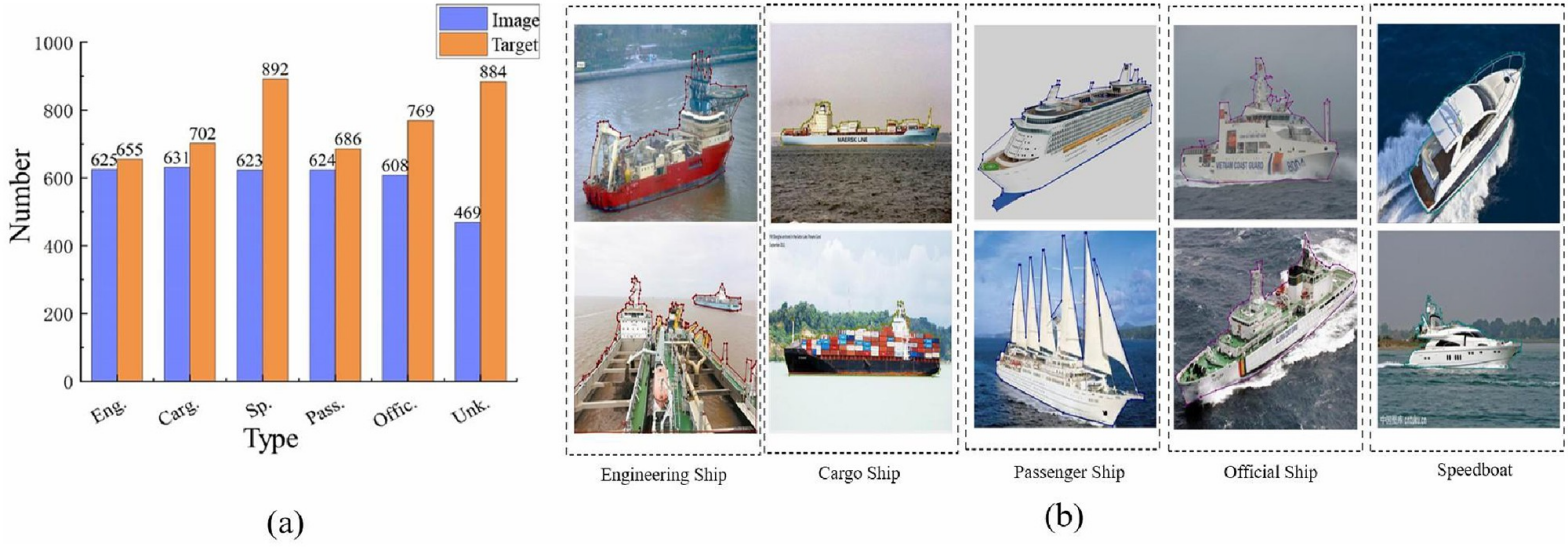
The MariBoatsSubclass dataset. (a) The histogram of the number of images and labelled ships in individual categories in MariBoatsSubclass. (b) Delineations of representative images of each category in MariBoatsSubclass.

To achieve a finer distinction of ship categories presented in individual images that can be used for ship instance segmentation, we constructed another dataset, namely MariBoatsSubclass, containing six categories as mentioned above. The dataset has 3125 high-quality VL images and 4588 labels. The number of labels are higher than that of VL images, which is because a single image may contain multiple ships of different types. Fig 6(a) shows the histogram of the number of ships in individual categories, among which the ‘Speedboat’ category has the highest number of images and labels (623 and 892, respectively). The category of ‘Unknown Ship’ has the lowest number of images, which is 469. In general, the distribution of each category and the accompanying segmented annotations are relatively evenly balanced, and the delineation of representatives of each category is shown in Fig 6(b).

### 2.3. Global and local attention mechanism

**Fig 7:**
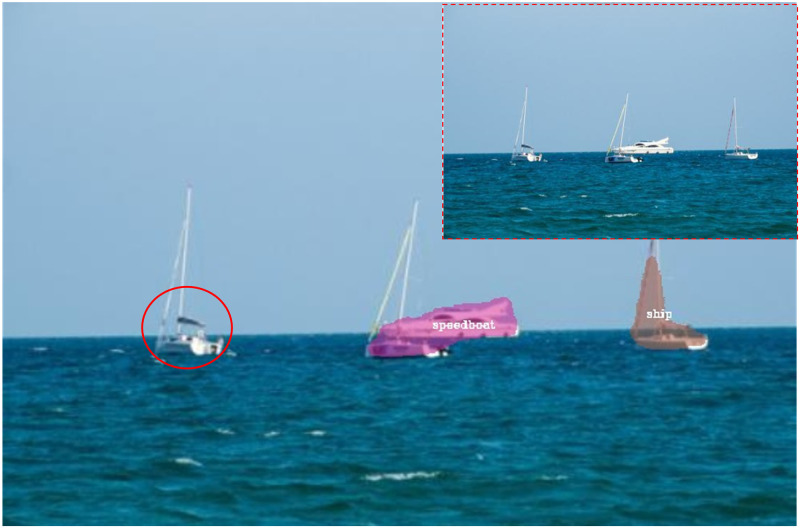
An example of a missed detection by the existing algorithms. The red circle represents the ship that should be detected but is not detected. Inset: the raw image.

After building two datasets for instance segmentation purpose, we next sought to test the performance of the existing instance segmentation models on our datasets. We tested classical models Mask R-CNN, SOLO and SOLOv2, and we found that the existing segmentation models to some extent cannot segment ships accurately because some ships were not detected by them (the red circle in Fig 7) [5, 39, 40]. The reason for the missed detection may due to the insufficient receptive field of the model, as discussed in [60], and the receptive field size of most CNN models is not proportional to its layer depth. An insufficient receptive field means that the global information of the inputs detected by a CNN model is not rich enough and one solution to this is to increase the receptive field. Pooling, a key element of CNN, is such a technique that can be adopted to increase the receptive field size of each convolutional kernel by downscaling the input graph, allowing the convolutional kernel to perceive a larger range of information from the input feature map.

**Fig 8:**
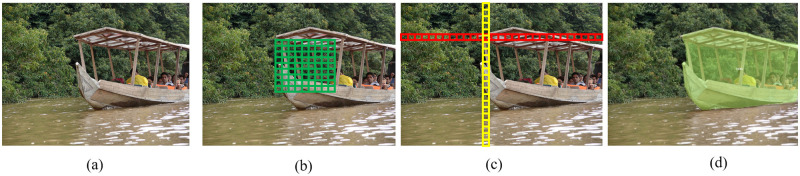
Illustration of the difference between 2D spatial pooling and 1D strip pooling in instance segmentation. (a) The original image. (b) 2-D spatial pooling. (c)1D strip pooling. (d) The segmentation result using the GALA mechanism.

Pooling can be further divided into 2D spatial pooling and 1D strip pooling, and the difference between how they work in instance segmentation is illustrated in Fig 8 [52]. 2D pooling is more “global” as it downscales and converts the 2D feature maps of a certain length and width into individual pixels (Fig 8b). With such a downscaling, the convolutional kernel of a neural network can focus on the global feature information of the inputs. 1D strip pooling applies dimensionality reduction in only one direction, by projecting the 2D feature map along the vertical or horizontal direction into one dimension. As shown in Fig 8c, the direction in yellow indicates that dimensionality reduction is applied along the vertical direction, while the horizontal direction remains unchanged (red). Similarly, dimensionality reduction can be applied along the horizontal direction (red), while keeping the vertical direction unchanged. In this way, the information in the feature map can be preserved locally and thus the convolutional kernel of the neural network can perceive the local information of an object. Intuitively, when applied to marine ship images, global feature information represents the relative spatial relationships between different classes of objects [61], and for example, the pixels representing the sky and the sea are mostly above and below the pixels of ships, respectively. This global spatial intra-class correlation can be used to improve the performance of CNN models for ship instance segmentation. If we further consider the relative local information such as that both sky and ship are located above the sea but the ship tends to be closer to the sea, this local information allows us to model more complex spatial relationships [62]. If a CNN model only considers local feature information, suppose that the CNN model detects a ship, then adding the local feature will only motivate the CNN to further detect the ships nearby, while for the ships located far away, the local feature will not take effect. Therefore, inspired by [61], in order to obtain a better segmentation performance than the existing CNN instance segmentation models aforementioned, we aimed to combine the global and local information.

To achieve this combination, we noticed that the global pooling mechanism is often implemented with the attention mechanism, an area under active investigation currently [43, 63], and we also noticed that these attention mechanism models mostly do not use the local feature information. In [48], a model using local pooling was proposed, but it did not use the global feature information. Here, in order to benefit from both the global and local pooling mechanism [61], we implemented the combination of the global and local mechanism together with the attention mechanism. By using both the global 2D spatial pooling and the local 1D strip pooling, we hoped that the new GALA mechanism is capable of improving segmentation performance. An example of the segmentation results by using the proposed GALA mechanism is shown in Fig 8d.

**Fig 9:**
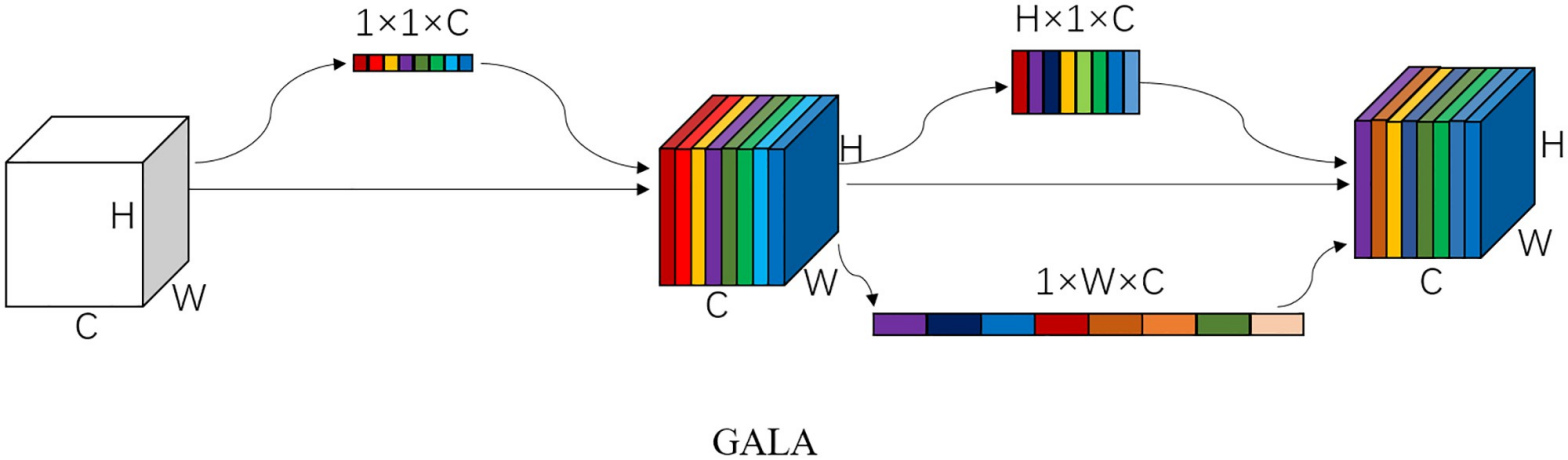
The scheme depicts the GALA mechanism. *C* represents the number of channels, that is, the number of feature maps.*H* and *W* represents the height and width of the feature maps, respectively.

The GALA mechanism was implemented as follows, schematically shown in Fig 9. First, we used the global average pooling to reduce the dimensionality of the entire 2D spatial information in a feature map, which is averaging all pixel values of each channel map, and we obtained a new 1 × 1 channel map. The output of the c-th channel feature map can be expressed as [43],

$$\begin{array}{c} z_{c}=\frac{1}{H\times W}\sum_{i=1}^{H}\sum_{j=1}^{W}x_{c}\left(i,j\right) \end{array}$$ (1)

where *x*_*c*_ is the input image of the c-th channel, *H* is the height of the input image, *W* is the width of the input image and *z*_*c*_ is the output image of the c-th channel.

After processed by the activation function and convolutional transformation, the feature map of channel correlation can be obtained and the output image of the c-th channel can be expressed as,

$$\begin{array}{c} {\hat{z}}_{c}=x_{c}\cdot \sigma \left(T_{2}\left(\delta \left(T_{1}\left(z_{c}\right)\right)\right)\right) \end{array}$$ (2)

where *σ* denotes the sigmoid function, and *T*_1_ and *T*_2_ are nonlinear transformations describing the importance of each channel. *δ* denotes the ReLU activation function. Second, we performed 1D pooling of the feature map with channel correlation in the horizontal and vertical direction respectively, and the output image of the c-th channel with height h can be expressed as,

$$\begin{array}{c} z_{c}^{h}\left(h\right)=\frac{1}{W}\sum_{0\leq i<W}{\hat{z}}_{c}\left(h,i\right) \end{array}$$ (3)

The output image of channel *c* with width *w* can be expressed as,

$$\begin{array}{c} z_{c}^{w}\left(w\right)=\frac{1}{H}\sum_{0\leq j<H}{\hat{z}}_{c}\left(j,w\right) \end{array}$$ (4)

where ${\hat{z}}_{c}$ denotes the input image of the c-th channel that already has channel correlation. After processing by the activation function and convolutional transformation, the final feature maps of channel correlation, direct perception, and position sensitivity can be obtained. The output image of the c-th channel can be expressed as,

$$\begin{array}{c} y_{c}\left(i,j\right)={\hat{z}}_{c}\times \sigma \left(F_{h}\left(z_{c}^{h}\left(h\right)\right)\right)\times \sigma \left(F_{w}\left(z_{c}^{w}\left(w\right)\right)\right) \end{array}$$ (5)

where *F*_*h*_ and *F*_*w*_ denote the two 1 × 1 convolutional transforms.

**Fig 10:**
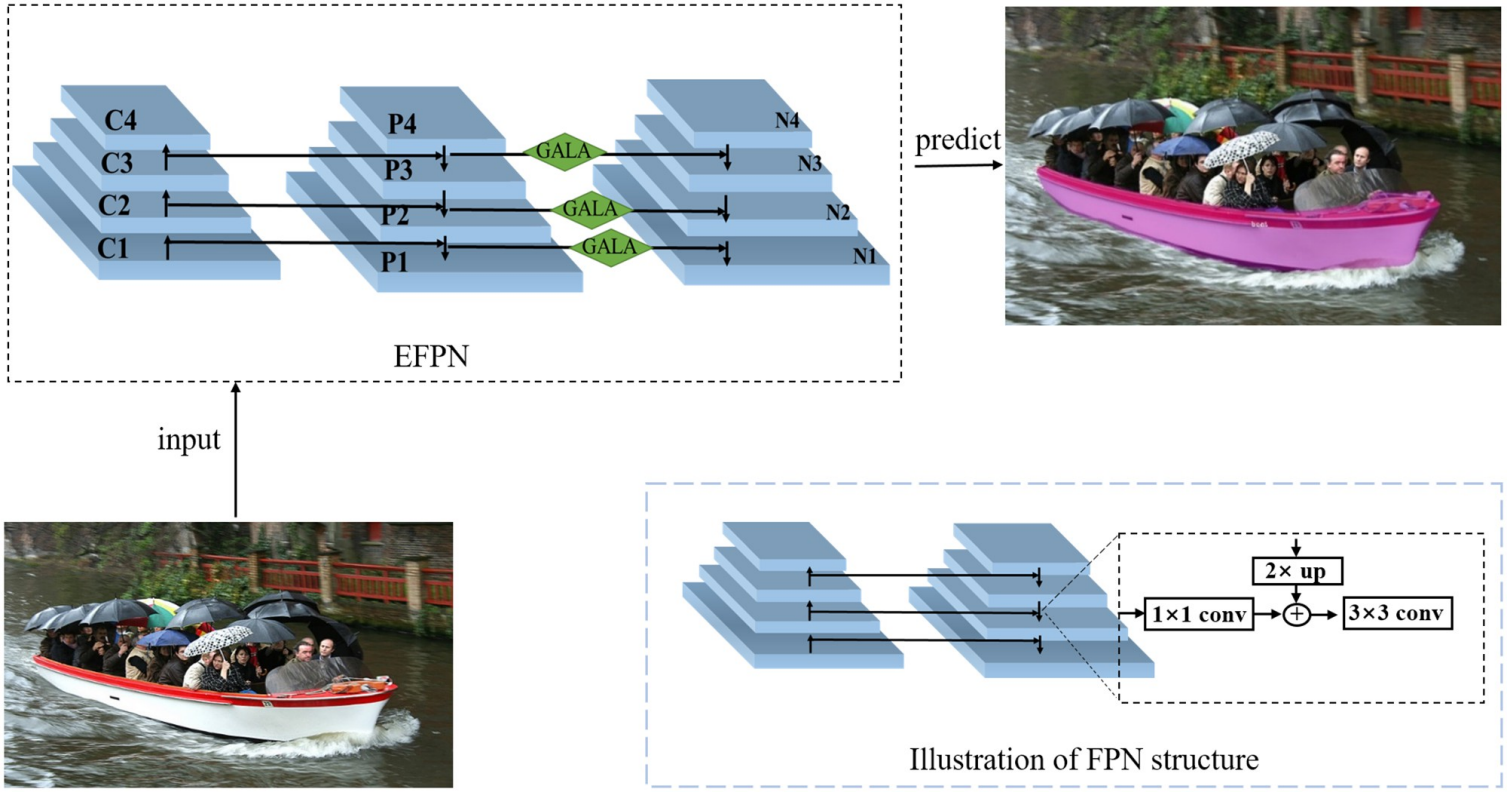
Schematic diagram of the enhanced feature pyramid structure. EFPN represents our enhanced improvement of the feature pyramid. GALA represents our proposed global and local attention mechanism. C1-C4 represent the feature maps of different sizes extracted from the backbone network, respectively, and P1-P4 represent the predicted objects of different scales, respectively. The original structure of the feature pyramid network is shown in the lower right.

In order to determine which layers of a neural network the GALA mechanism is specifically applied to, we deployed GALA into a feature pyramid network containing multi-scale feature information. The shallow feature maps of the feature pyramid network characterize the detailed information of an object and the deep feature maps characterize the semantic information of an object. The combination of feature maps with different depths within the network forms multi-scale representation information. To take full advantage of the multi-scale representation in the feature pyramid network, here we propose an Enhanced Feature Pyramid Network (EFPN) based on the GALA mechanism, as shown in Fig 10. For each layer of the different scale predictions of the feature pyramid network, the GALA mechanism is used to enhance the representation of the feature graph.

## 3 Experiment results

Transfer learning is a technique to avoid training a neural network from scratch, and here we used the pre-trained ImageNet model as the start point for retraining using our datasets [64]. The experimental environment was configured with Ubuntu 16.04.4, pytorch1.4.0, and 4 NVIDIA GeForce GTX 1080Ti GPUs. The learning rate is 0.01. The optimization algorithm is the Stochastic Gradient Descent. We first tested the performance of several classical instance segmentation models, namely Mask R-CNN, SOLO and SOLOv2 models, on our datasets which included coco_boats, self_boats, MariBoats (coco_boats + self_boats), and MariBoatsSubclass. The datasets and accompanying algorithms can be download via the link, https://github.com/s2120200252/Visible-ship-dataset.

### 3.1 Performance evaluation metrics

We followed the quantitative metric system used by the MS COCO dataset to evaluate the performance of ship instance segmentation models. The six metrics used are Intersection of Union (IoU), Average Precision (AP), Mean Average Precision (mAP), Frames Per Second (FPS), Parameter (Para.) and Time Complexity (TC). We refer to a more detailed definition about these metrics [58]. IoU is defined as the degree of overlap between two segmentations. AP is the major metric that will be used for accuracy determination.

$$\begin{array}{c} AP=\int_{0}^{1}P(R)\text{d}R, \end{array}$$ (6)

where P represents precision and R represents recall rate. AP calculates an average IoU, averaging from 0.5 to 0.95 with increment of 0.05. For example, AP50 and AP75 represent the calculation of the average IoU at thresholds of 0.5 and 0.75, respectively. For multi-scale object detection capability, *AP*_*S*_, *AP*_*M*_, and *AP*_*L*_ are used to represent the average accuracy of objects of small (area < 32^2^ pixels), medium (32^2^ < area < 64^2^ pixels), and large (area > 64^2^ pixels) size, respectively. Mean Average Precision(mAP) is a mean value of AP over the number of categories of ships to be detected. In addition to evaluate the accuracy, it is also important to compare the running time of the tested instance segmentation models. FPS is such a metric that measures the number of images a model can process in a second. Parameter is the number of parameters in a neural network model (the parameters learned when training the network).

$$\begin{array}{c} P\text{arams}.=C_{o}\times \text{(}k^{2}\times C_{i}), \end{array}$$ (7)

where *C*_*o*_ represents the number of input channels, *k* represents the size of a convolution kernel, and *C*_*i*_ represents the number of output channels. Generally speaking, the number of parameters is positively proportional to the memory required to save the model and the hardware memory requirement. The metric Memory Access Cost (MAC) is generally used to measure the time complexity (TC) of a model or an algorithm, defined as,

$$\begin{array}{c} TC=2\times C_{i}\times k^{2}\times C_{o}\times W\times H, \end{array}$$ (8)

where *C*_*o*_ represents the number of input channels, *k* represents the size of a convolution kernel, and *C*_*i*_ represents the number of output channels, *W* represents the width of a feature map, *H* represents the height of a feature map.

### 3.2 Performance test

#### 3.2.1 Segmentation results on MariBoats having one ship category

We next moved onto the performance evaluation of the existing models, i.e. Mask R-CNN, SOLO and SOLOv2, on our datasets. We first performed the evaluation on the coco_boats dataset, and we found that the segmentations of the images in coco_boats by all the three models were not satisfactory (AP: 9.4,13.4,13.6), which are mostly due to the fact that these models have not been trained on enough VL ship images. Actually, the poor performance of these models on coco_boats was our initial motivation for building a larger dataset, containing sufficient VL images of marine ships, and we hoped that retraining the models by this larger dataset, MariBoats, can improve the performance of these models. We then used 70% of MariBoats to retrain the three models aforementioned, and used the remaining 30% for testing. The results of the quantitative tests are shown in Tables 2–4, which directly indicate that the segmentation accuracy of the three network models has improves for both MariBoats and the two subsets of MariBoats, coco_boats and self_boats, after retraining the models by MariBoats (the last row in Tables 2–4).

**Table 2:** Test results of Mask-RCNN in three instance segmentation datasets.

Mask R-CNN	coco_boats_test/(AP)	self_boats_test/(AP)	MariBoats_test/(AP)
coco_boats_train	9.5	22.4	18.1
self_boats_train	4	53.3	37.7
MariBoats_train	**10.8**	**55.6**	**42.4**

**Table 3:** Test results of SOLO in the three instance segmentation datasets.

SOLO	coco_boats_test/(AP)	self_boats_test/(AP)	MariBoats_test/(AP)
coco_boats_train	13.6	35.2	28.3
self_boats_train	5.3	59.2	42.2
MariBoats_train	**14.9**	**61.2**	**47.2**

**Table 4:** Test results of SOLOv2 in the three instance segmentation datasets.

SOLOv2	coco_boats_test/(AP)	self_boats_test/(AP)	MariBoats_test/(AP)
coco_boats_train	13.4	32.5	26.4
self_boats_train	5	59.6	42.5
MariBoats_train	**15.8**	**65.3**	**50.4**

Meanwhile, we further tested the segmentation accuracy of the three models by using only coco_boats or self_boats to retrain the models and using the other two datasets for testing (row 1-2 in Tables 2–4). Taking the SOLOv2 as an example (Table 4), the segmentation accuracy of coco_boats shows that the model trained on the MariBoats training set improves by 2.4% compared with the results trained on the coco_boats training set. The results of self_boats show that the model trained on the MariBoats training set improves by 5.7% compared with the results trained on the self_boats training set. The MariBoats results show that the model trained on the MariBoats training set improves by 7.9% compared with the results trained on the self_boats training set. Tables 2 and 3 further verify the advantages of the MariBoats dataset, containing richer image data, in improving the segmentation performance of Mask R-CNN and SOLO, respectively. Taken together, these data fully validated the necessity of building the self_boats dataset and the MariBoats dataset.

#### 3.2.2 Segmentation results on MariBoatsSubclass having six ship categories

**Fig 11:**
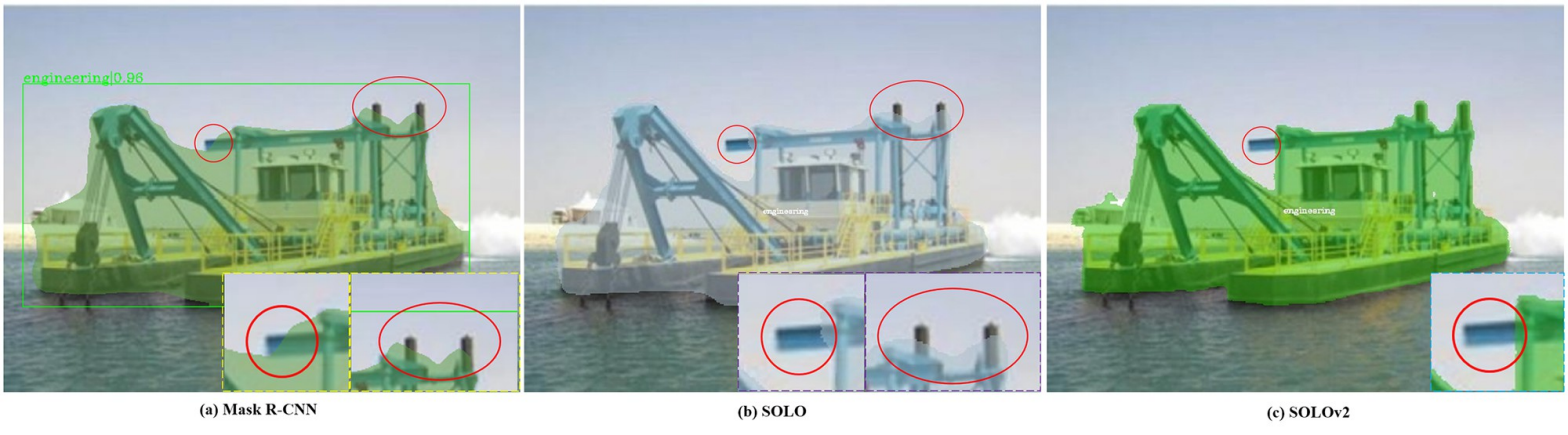
A representative example of incomplete segmentation by Mask R-CNN, SOLO and SOLOv2. The red circles represent the parts of ships that should be detected but are not detected. Insets: amplified images of the objects with the red circles.

We continued to test the segmentation performance and computational speed of Mask R-CNN, SOLO, and SOLOv2 on MariBoatsSubclass upon solving an instance segmentation problem having six ship categories. The experimental results are shown in Table 5. We first set the backbone network ResNet to commonly used 50 layers for Mask R-CNN, SOLO, and SOLOv2 (row 1-3 in Table 5), and SOLOv2 had the highest score in terms of mAP (57.8%), compared with 42.9% and 55.5% for Mask R-CNN and SOLO, respectively. The segmentation accuracy of these models on segmenting the Unknown ship category is generally much lower compared with other categories, and we reasoned that the smaller size of the ships (having fewer pixels) shown in this category can affect the evaluation of the IoU metric. However, the mAP of all six-class ships is higher. These further validated the diversity of ship scales in MariBoatsSubclass. We also noticed that although these model showed improvement after retraining by MariBoatsSubclass, there is still quite some room to improve the performance of these models. For example, these model are still suffering from incomplete segmentation, as shown in Fig 11.

**Table 5:** Test results of different network structures on MariBoatsSubclass.

Method	mAP	Eng./(AP)	Carg./(AP)	Sp./(AP)	Pass./(AP)	Offic./(AP)	Unk./(AP)	FPS
Mask R-CNN(Resnet-50)	42.9	43.11	56.12	39.24	54.12	52.16	12.89	16
SOLO(Resnet-50)	55.5	62.44	71.53	53.68	67.99	62.13	**15.52**	28.3
SOLOv2(Resnet-50)	**57.8**	**68.93**	**76.14**	**51.71**	**69.92**	**64.54**	15.33	33
SOLOv2(Resnet-18)	56.2	68.26	73.90	50.38	68.91	61.32	14.66	**44.7**
SOLOv2(Resnet-34)	57.4	68.22	75.77	52.45	69.39	62.66	15.83	39.9

Since SOLOv2 had the best performance, and we then selected SOLOv2 to test the computational speed using a different number of layers (row 4-5). The segmentation accuracies for SOLOv2 were 56.2% and 57.4% in terms of mAP when the backbone network ResNet was set to 18 and 34 layers, respectively. Although the mAP values decreased by 1.6% and 0.4%, respectively, the computational speed of SOLOv2 improved by 35% and 20% in terms of FPS, reaching 44.7 and 39.9, respectively. This suggests that the segmentation speed can be significantly improved with a small loss of AP by reducing the number of residual layers of the backbone network.

### 3.3 Performance testing of the proposed GALA mechanism

Based on the segmentation results of the MariBoatsSubclass dataset, SOLOv2 achieved the highest segmentation accuracy and was selected as the base network to implement the attention mechanism. Note that the attention mechanism has not been used in the existing models including Mask R-CNN, SOLO, and SOLOv2. Here, we not only introduced the attention mechanism into SOLOv2, and we further proposed a new attention mechanism, GALA, to improve the performance of the existing attention mechanisms. To prove the performance of the proposed GALA mechanism, we introduced different classical attention mechanisms to SOLOv2 as a comparison (Table 6). As shown in Table 6, compared with SOLOv2 without using any attention mechanism, the performance of all SOLOv2 models using several classical attention mechanisms ECA-Net [65], ScSE [63], Triplet Attention [66], SENet [43], and CA [48], improved in terms of mAP, and the one using GALA mechanism had the largest improvement, reaching 62.1%, which is 4.3% improvement with respect to SOLOv2 and 3.8%, 3.6% and 3.3% improvements with respect to CA, Triplet Attention and SEnet, respectively. This validated the performance of the proposed GALA attention mechanism over the existing attention mechanism, on the VL ship datasets.

**Table 6:** The performance of different attention mechanisms on MariBoatsSubclass.

Methods	mAP	Eng./(AP)	Carg./(AP)	Sp./(AP)	Pass./(AP)	Offic./(AP)	Unk./(AP)	FPS	Para./M	TC/(GMAC)
SOLOv2	57.8	68.93	76.14	51.71	69.92	64.54	15.33	**33**	**30.94**	**58.12**
ScSE [63]	58.3	69.64	76.47	52.49	71.37	64.31	15.35	32.2	31.01	58.14
CA [48]	58.3	68.94	76.10	53.00	69.78	64.87	17.28	31.4	30.95	58.15
ECA-Net [65]	58.4	69.35	76.74	53.62	69.95	64.84	16.075	32.7	30.94	58.13
Triplet Attention [66]	58.5	69.86	77.42	52.98	70.24	64.72	15.73	32.2	30.94	58.14
SENet [43]	58.8	69.64	77.54	53.49	70.74	69.07	16.54	32.7	30.95	58.13
+GALA(ours)	**62.1**	**70.24**	**79.16**	**60.28**	**73.44**	**69.41**	**20.31**	31.4	30.96	58.15

We further analyzed the computational complexity of the SOLOv2 model using GALA, in terms of the increase in the number of parameters and FPS. The increase in the number of parameters with respect to SOLOv2 was only 0.02 M. This increase is nearly negligible compared with the 30.94 M parameters of SOLOv2. The increase in time complexity was only 0.03 GMAC, and the FPS decreased by only 1.6. This analysis indicates that introducing GALA into the feature pyramid network can improve the performance of instance segmentation models significantly with little increase in time complexity and number of parameters.

**Fig 12:**
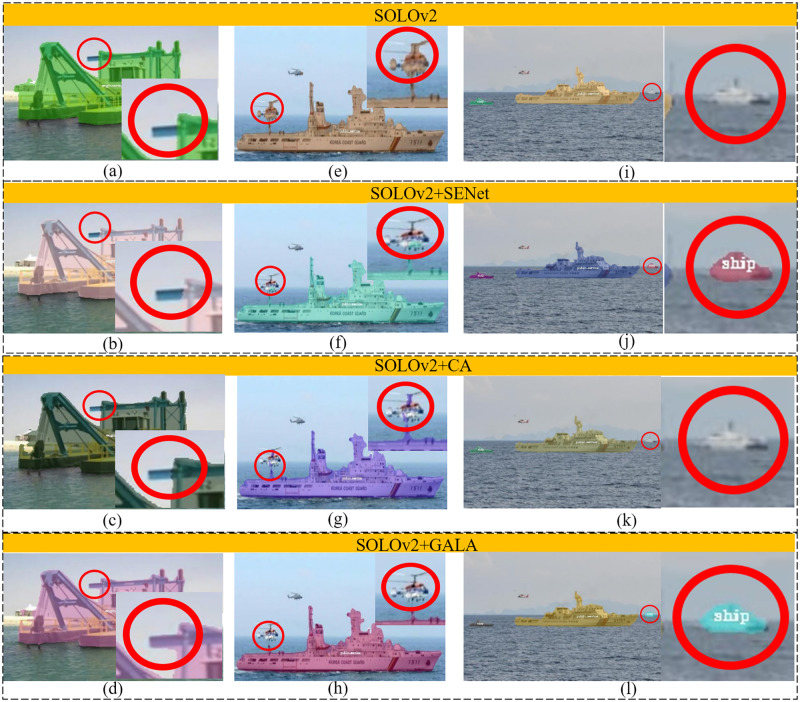
Comparison of segmentation results for several typical ship examples, selected from MariBoatsSubclass. The first row (a, e, i): segmentations of SOLOv2. The second row (b, f, j): the segmentations of SOLOv2+ SEnet. The third row (c, g, j): the segmentations of SOLOv2+ CA. The last row (d, h, l): the segmentations of SOLOv2 using the proposed GALA mechanism.

Visually, GALA also improved the segmentation performance of SOLOv2 on MariBoats. As shown in Fig 12, SOLOv2, SOLOv2+SEnet, and SOLOv2+CA networks cannot detect completely all the ships, by either missing a ship partially (the red circles in Fig 12a–12c) or entirely (Fig 12i and 12k). In addition, SOLOv2, SOLOv2+SEnet, and SOLOv2+CA networks incorrectly detected the aircraft as a part of the ship below it (Fig 12e–12g). However, SOLOv2+GALA was able to correctly separate the aircraft from the ship, and could detect all the ships completely and correctly (Fig 12d, 12l, and 12h). Therefore, this visual comparison together with the quantitative comparison verified the superiority of the proposed GALA mechanism over the existing attention mechanisms.

## 4 Conclusion and discussion

Marine ship instance segmentation of VL images plays an important role in marine-related scientific research, educational and commercial applications. However, there are hardly any publicly available and suitable datasets containing VL images of marine ships for ship instance segmentation purpose. To address this, we collected and manually labelled two new VL marine ship datasets using a data collection tool that we developed. The datasets and the accompanying image processing tools are available to the public for visual perception applications of marine scenes. To the best of our knowledge, there are also rare instance segmentation methods that are specially designed for marine ship segmentation. Therefore, considering the special characteristics of marine ship instance segmentation, we proposed the GALA attention mechanism, which takes advantage of 1D strip pooling and 2D spatial pooling to preserve both the global and local information of the input images. Experimental results demonstrated the superiority of GALA over the existing attention mechanisms on the marine ship datasets. We selected Mask R-CNN, SOLO, and SOLOv2 as comparison because these models have been well recognized in the computer vision and remote sensing fields. Many works have tried to improve these models, such as SOLO series models [39, 40], and the Cascade R-CNN models [67], and these variants are also widely applied in many fields.

Sun et al. has built a VL dataset, MariShipInsSeg, which contains 4K images but MariShipInsSeg is not open-source and this dataset has one general ship category [16]. In contrast, our dataset MariBoats has more images and is open-source. In addition, we have further refined the category of MariBoats into six ship categories, leading to our second dataset, MariBoatsSubclass. It will facilitate the research on accurate segmentation and classification of marine ships based on appearance, shape and function. Moreover, we have introduced the attention mechanism into the marine ship instance segmentation of VL images and we have further proposed a novel attention mechanism which can improve the performance of existing neural networks. It is important to notice that although the attention mechanism has been applied to the COCO dataset before, there are no such attention models specially designed for marine ship instance segmentation of VL images. Marine ship images are different from most of the images in the COCO dataset in terms of background, texture, and contour of objects. Therefore, it is the first time that the attention mechanism is introduced into marine ship instance segmentation using VL images. Most of the existing attention mechanisms such as ECA-Net [65], ScSE [63], Triplet Attention [66], and SENet [43], have focused on the global information, and only a few attention mechanisms such as CA [48], have paid attention to the local information. Inspired by [61], we combined both the global and local mechanism to retain both the global and local feature information, achieving better segmentation results than the existing attention mechanisms using only global or local information alone. Our proposed GALA mechanism maintains the convenience of the attention mechanism that can be applied generally to most of the neural networks, and the source codes are immediately available to the public. Finally, we have also made the accompanying data extraction and analysis tools publicly available to facilitate research in the computer vision field.

In conclusion, we believe that the new open-source datasets we built in this work and the proposed GALA mechanism will facilitate research in VL ship applications and attract attention from other computer vision fields to use the GALA mechanism. Future work includes enriching the current datasets for marine ship instance segmentation and developing fast segmentation methods for segmenting ships from complex ocean scenes. Moreover, we also believe that the proposed mechanism is applicable to other fields such as remote sensing and biomedical microscopic data, because the instant segmentation of targets in these datasets, for example marine ships in SAR image datasets [57], and cells in the microscopic datasets [68, 69], also relies on the use of global and local information to describe the targets and to distinguish the targets from the backgrounds. The application of the GALA attention mechanism to these datasets will be a valuable plan for future to explore.
